# Supplementary material for: Synthesis, Anticancer, Antioxidant, Anti-Inflammatory, Antimicrobial Activities, Molecular Docking, and DFT Studies of Sultams Derived from Saccharin
Source: Molecules. 2022 Oct 20;27(20):7104. doi: 10.3390/molecules27207104 (PMC9611055; doi:10.3390/molecules27207104)
Supplement: Supplementary file 1 [file molecules-27-07104-s001.zip › molecules-1929135-supplementary.pdf]

Supplementary Materials

Synthesis, Anticancer, Antioxidant, Anti-inflammatory, Antimicrobial Activities, Molecular Docking, and DFT Studies of Sultams Derived from Saccharin

Nourah Al-Fayez [1], Hany Elsawy [1,2], Mohammed A. Mansour [2,3], Mohamad Akbar Ali [1,4] and Ibrahim Elghamry [1,*]

1 Department of Chemistry, College of Science Al Hufuf, King Faisal University, P.O. Box 380, Saudi Arabia
2 Biochemistry Division, Department of Chemistry, Faculty of Science, Tanta University, Tanta 31527, Egypt
3 Cancer Biology and Therapy Lab, Division of Human Sciences, School of Applied Sciences, London South Bank University, London SE1 0AA, UK
4 Department of Chemistry, College of Art and Science, Khalifa University, 127788_Abu Dhabi, United Arab Emirates
* Correspondence: ielghamry@kfu.edu.sa or elghamry@hotmail.com; Tel.: +966-135-899-557

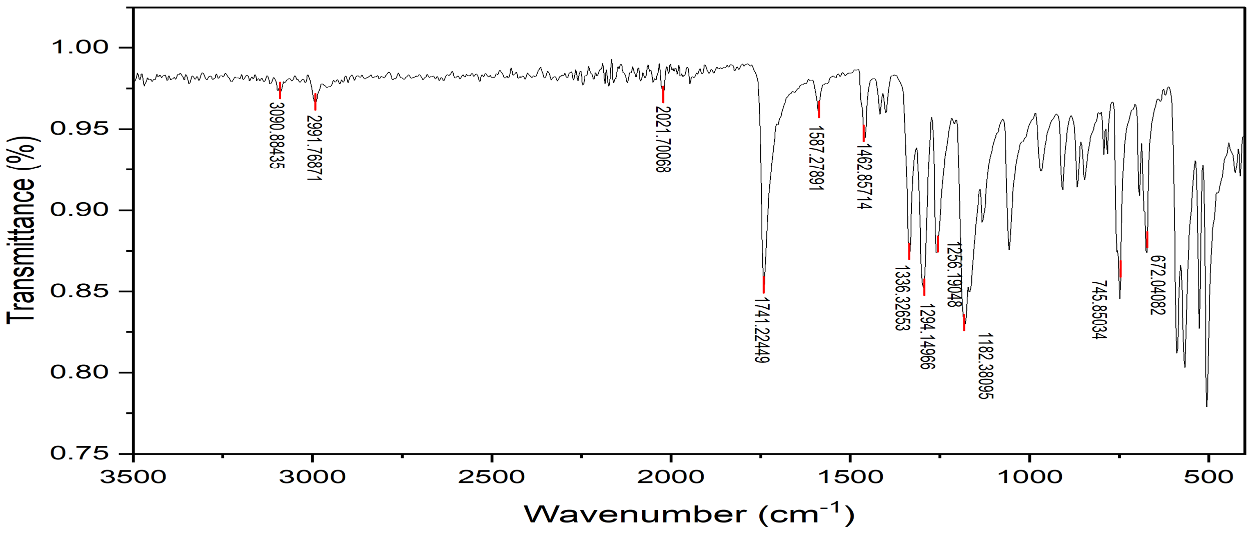

Figure S1. IR spectra of compound **2**.

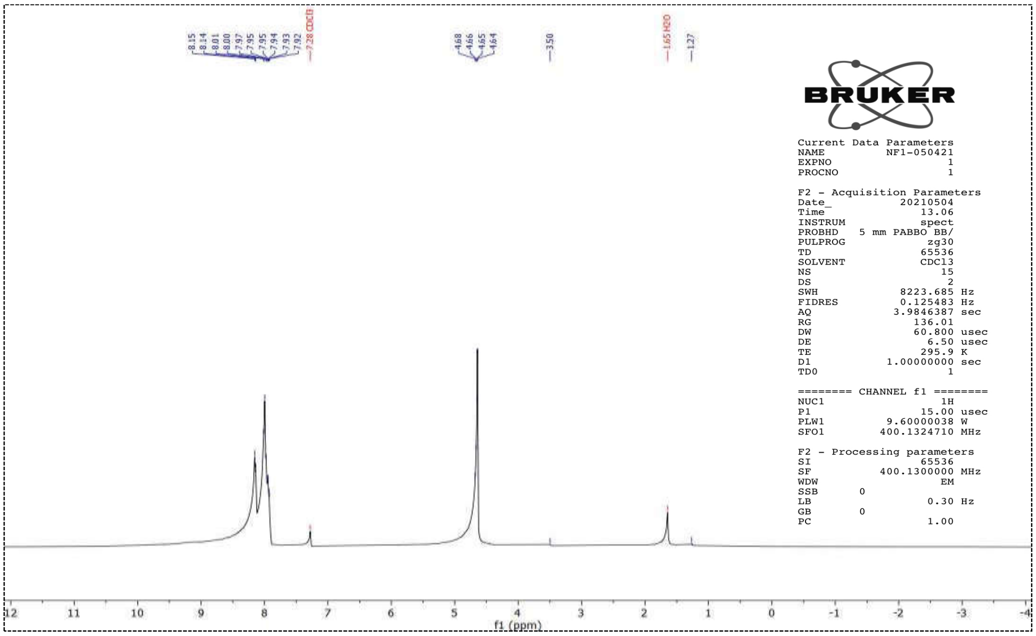

Figure S2. ^{1}H NMR spectra of compound **2**.

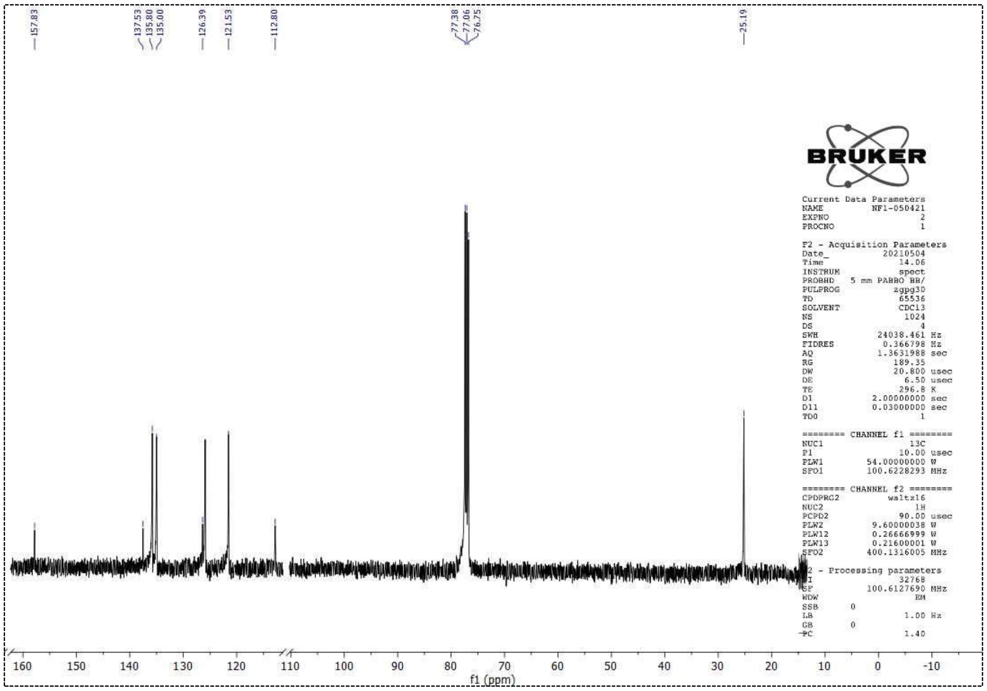

Figure S3. ^{13}C NMR spectra of compound **2**.

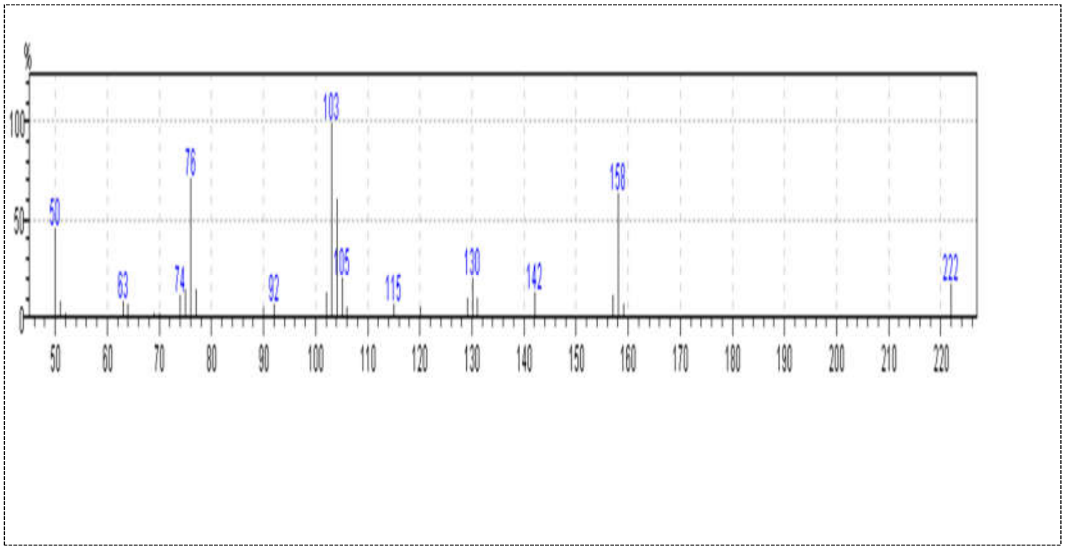

Figure S4. MS spectra of compound **2**.

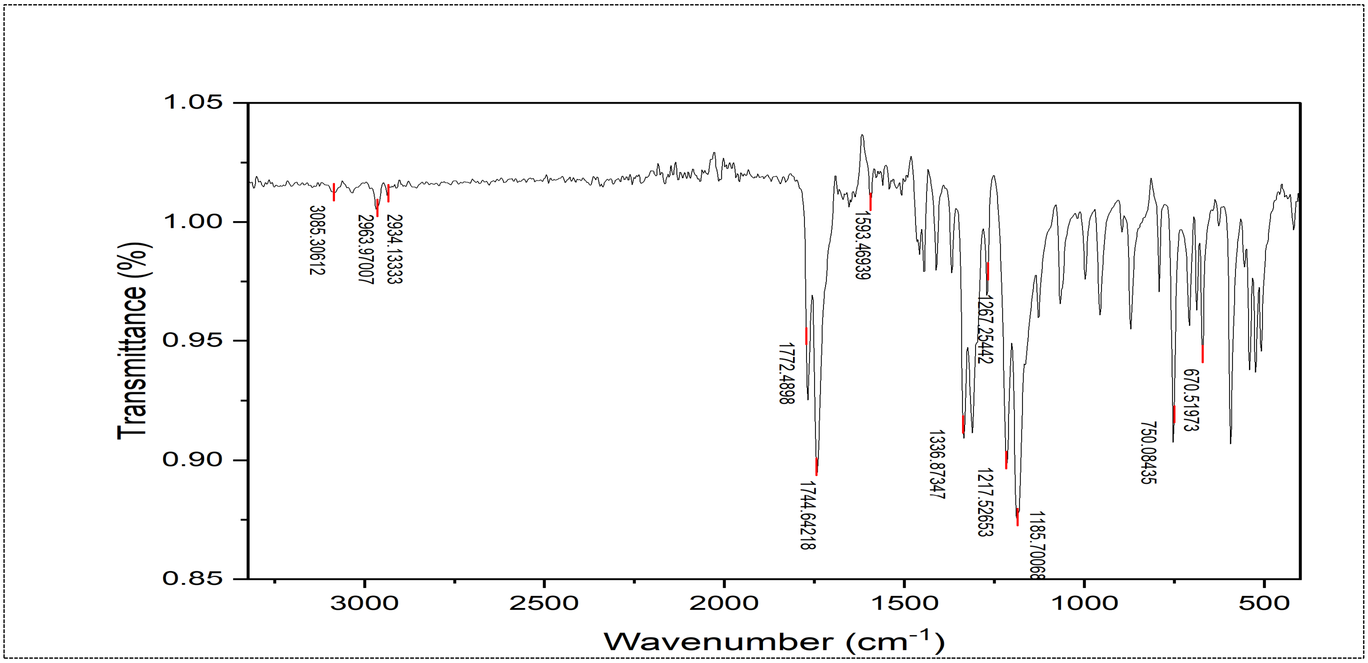

Figure S5. IR spectra of compound **3a**.

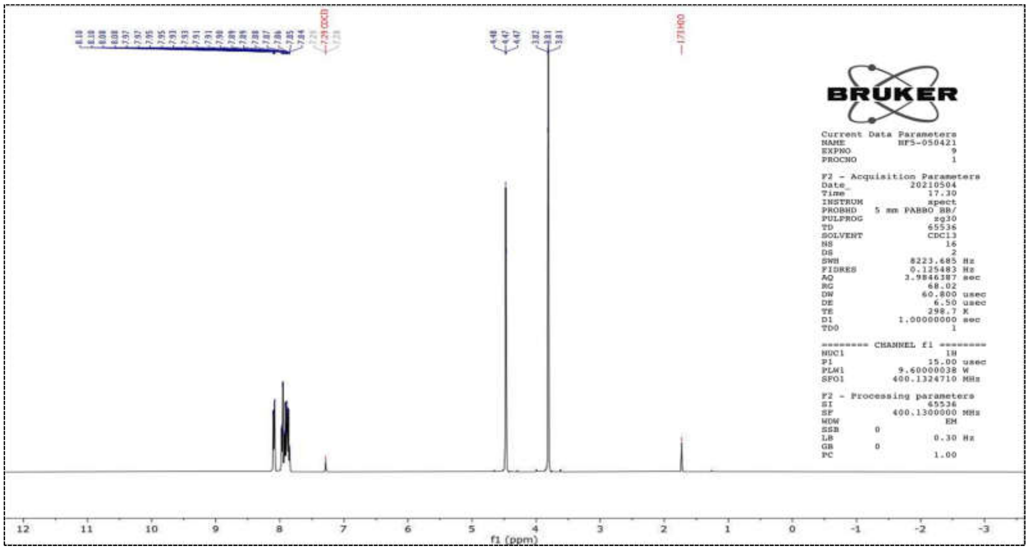

Figure S6. ^{1}H NMR spectra of compound **3a**.

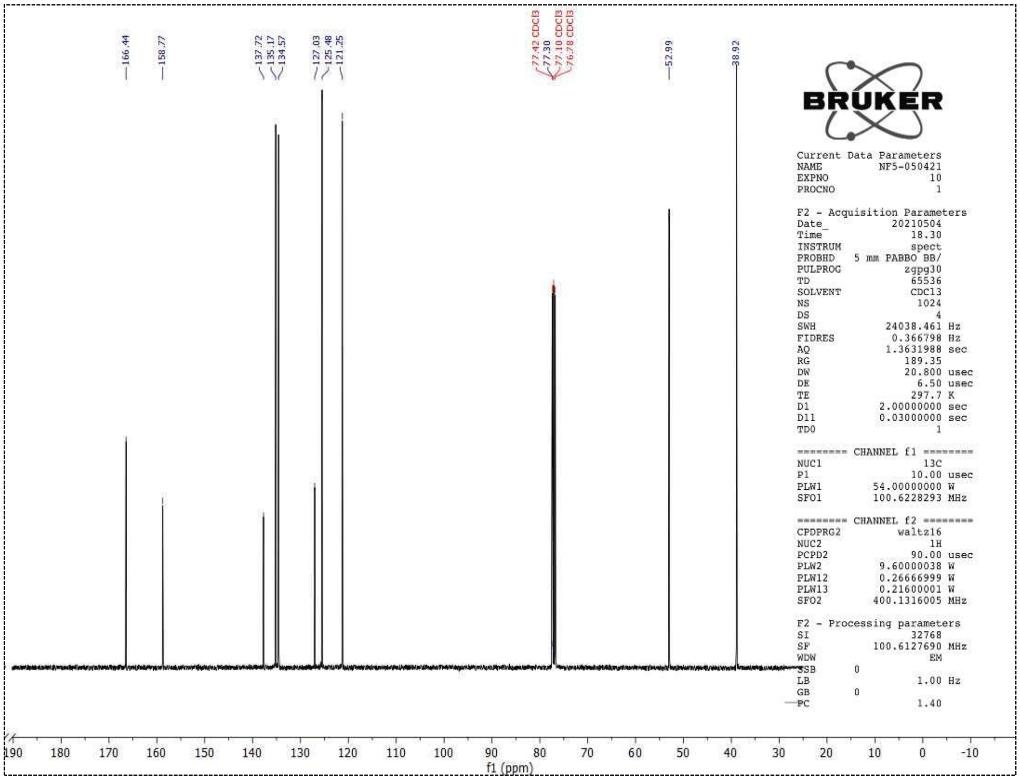

Figure S7. ^{13}C NMR spectra of compound **3a**.

Chemical Formula: $C_{10}H_9NO_5S$

Molecular Weight: 255.25 g/mol

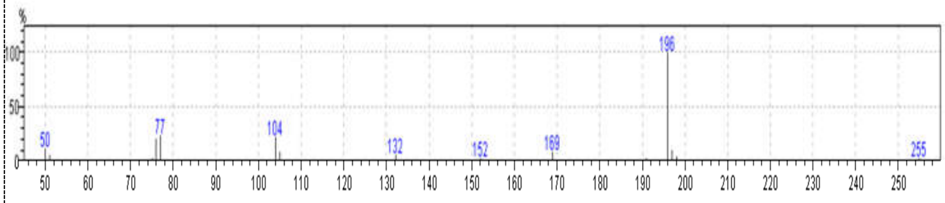

Figure S8. MS spectra of compound **3a**.

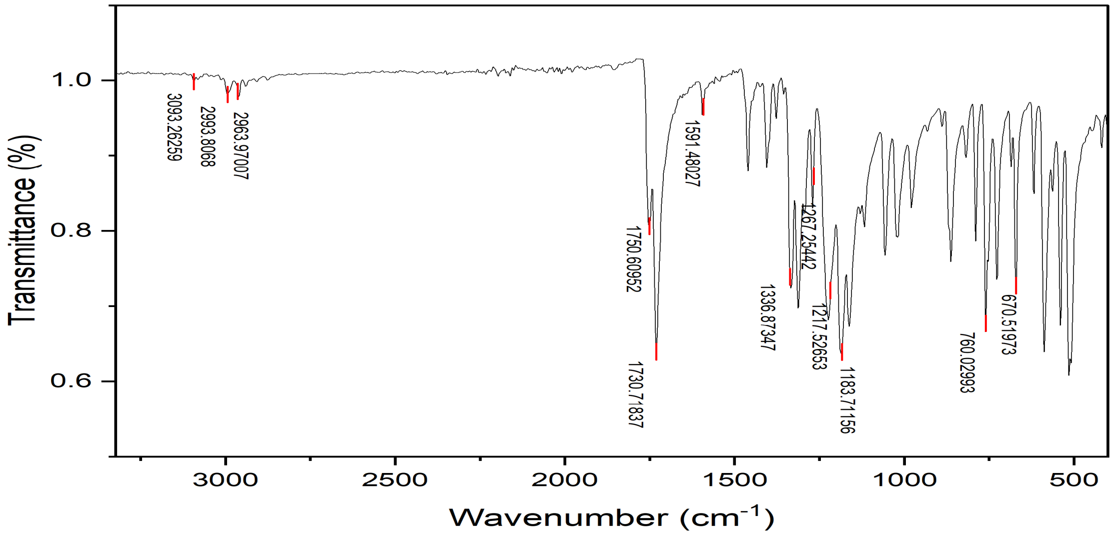

Figure S9. IR spectra of compound **3b**.

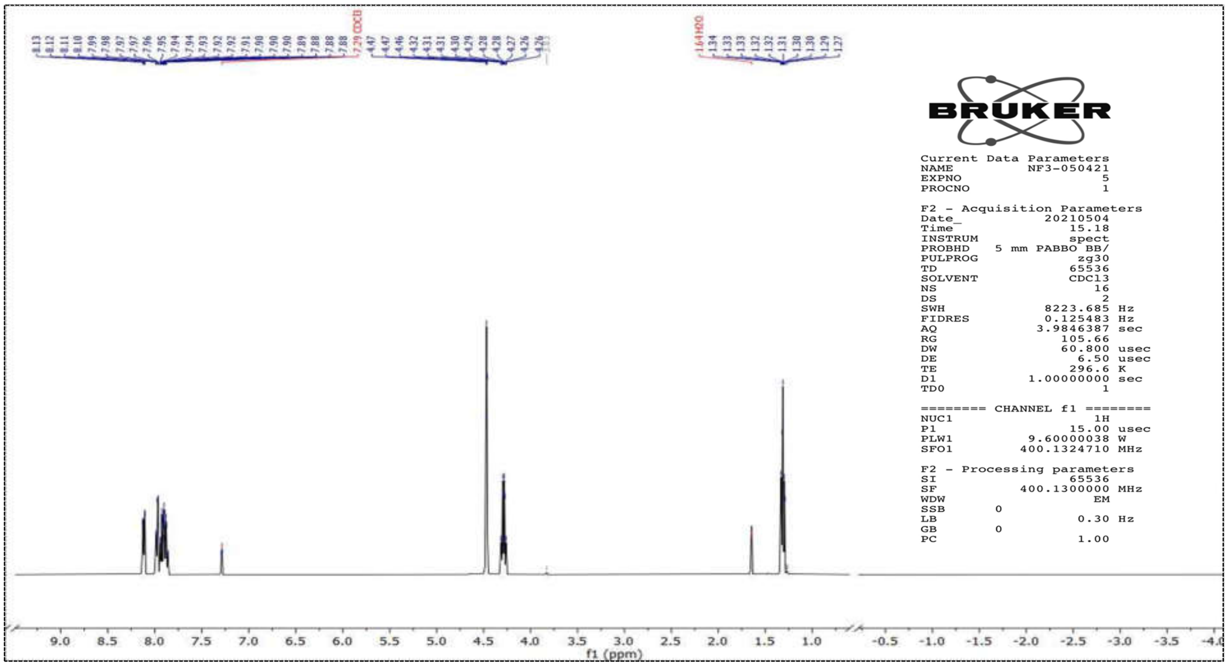

Figure S10. ^{1}H NMR spectra of compound **3b**.

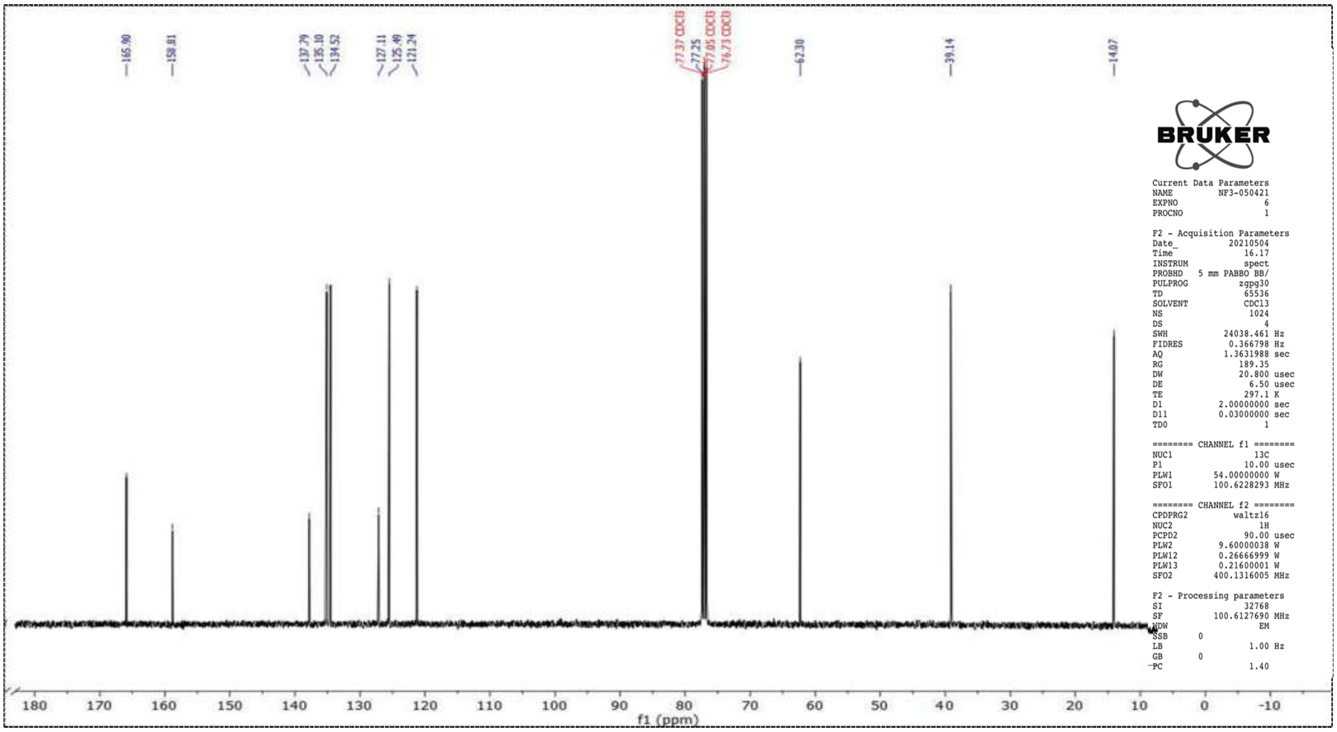

Figure S11. ^{13}C NMR spectra of compound **3b**.

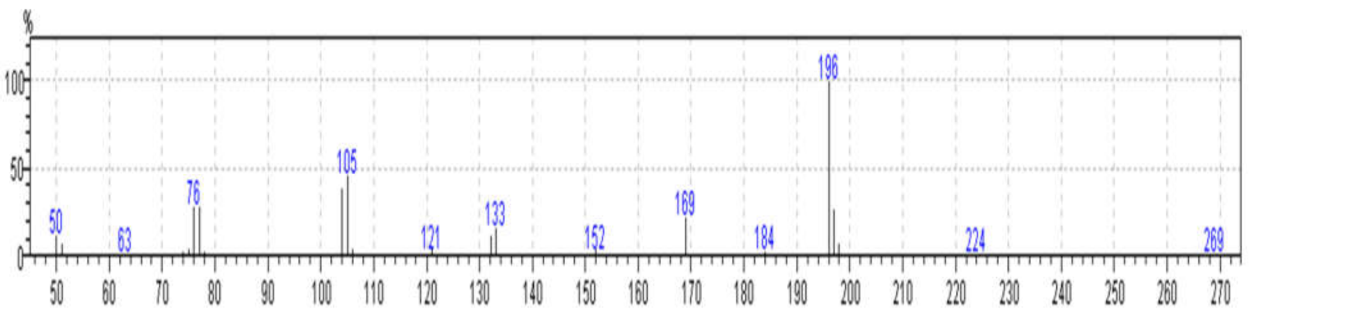

Figure S12. MS spectra of compound **3b**.

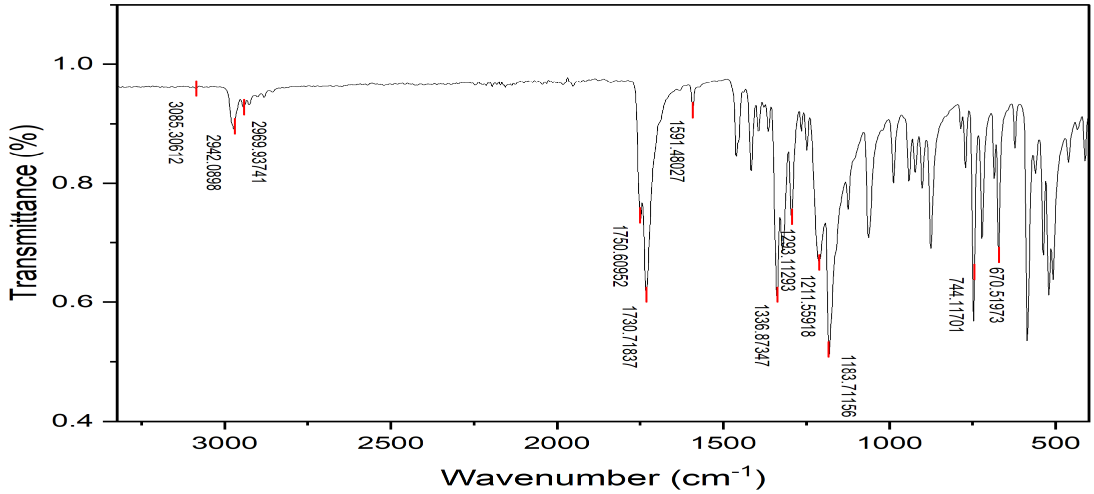

Figure S13. IR spectra of compound **3c**.

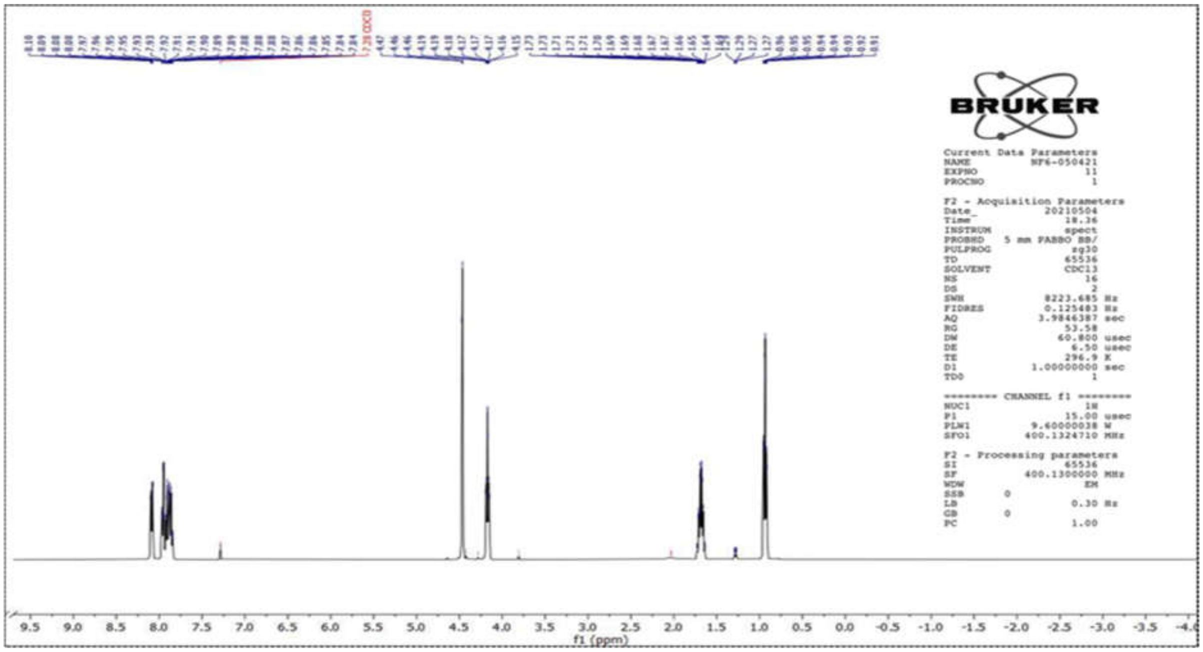

Figure S14. ^{1}H NMR spectra of compound **3c**.

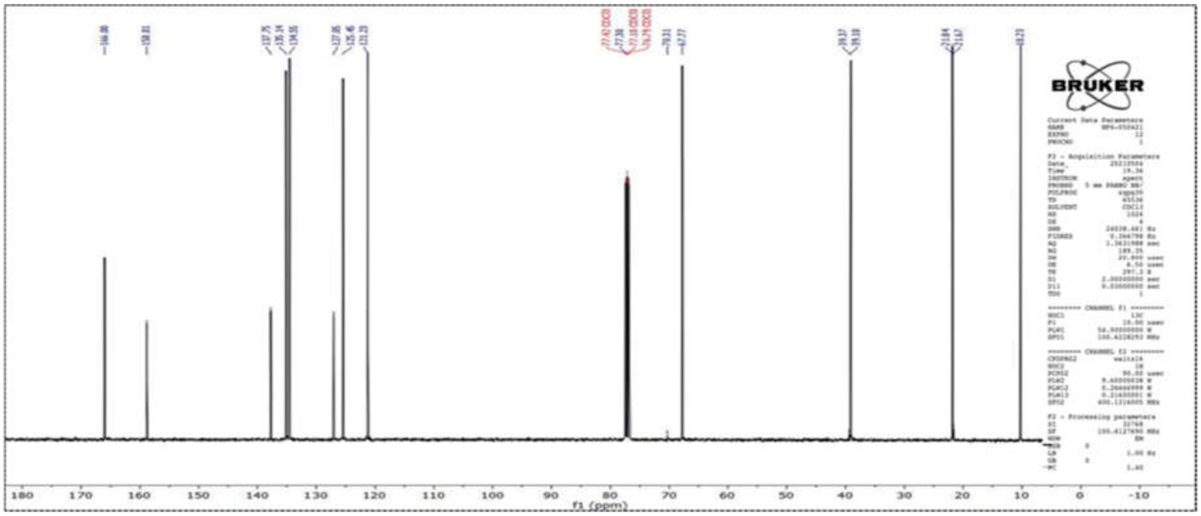

Figure S15. ^{13}CNMR spectra of compound **3c**.

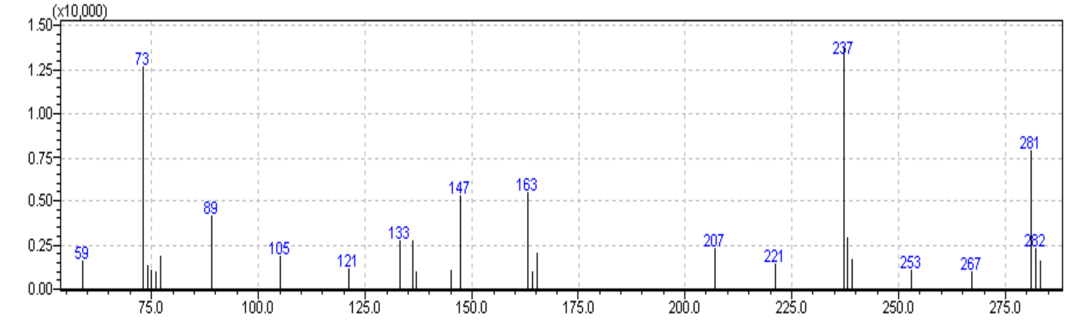

Figure S16. MS spectra of compound **3c.**

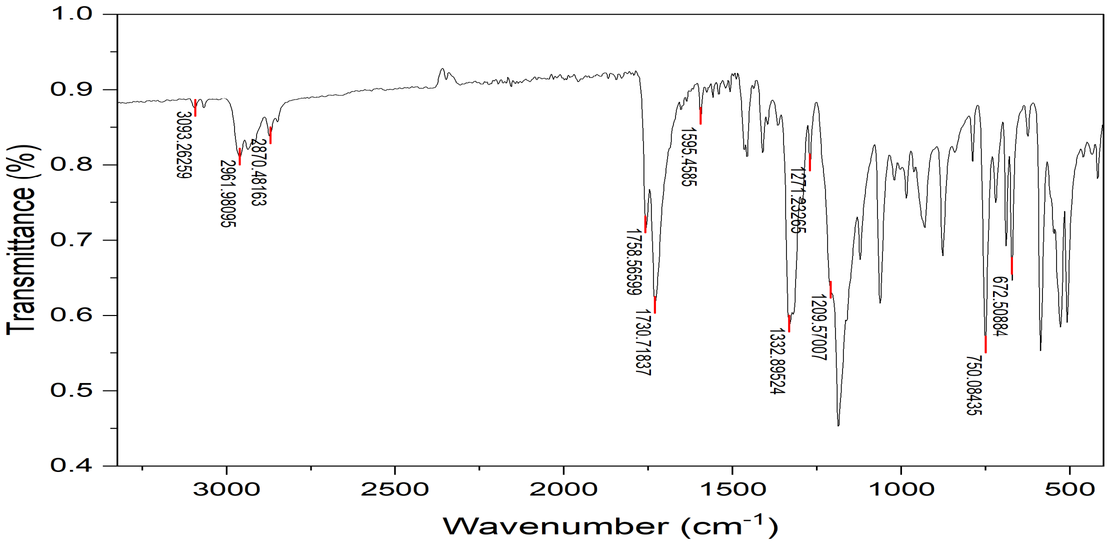

Figure S17. IR spectra of compound **3d.**

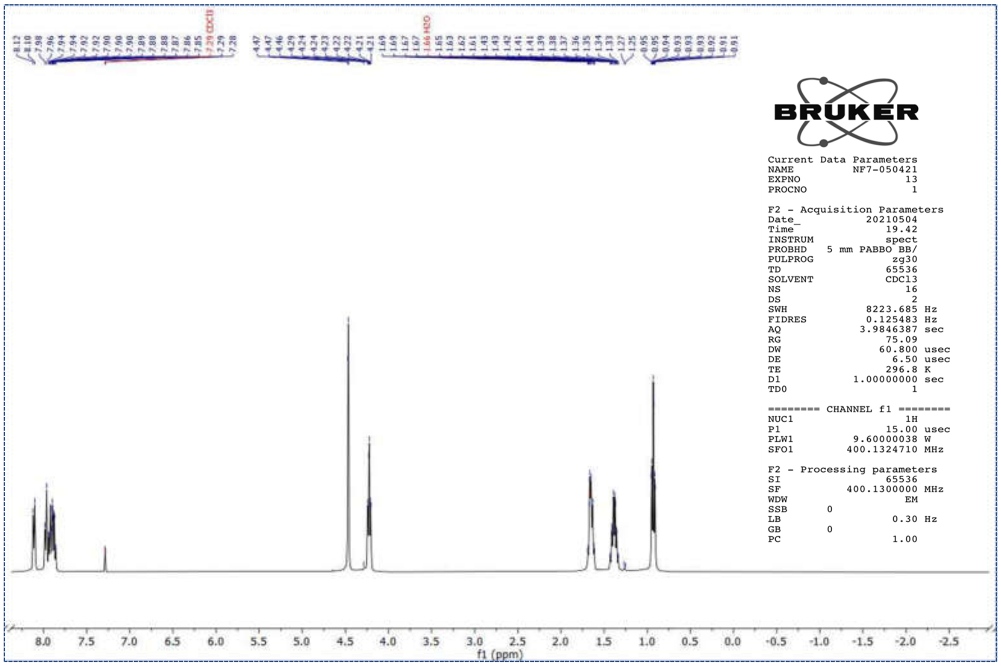

Figure S18. ^{1}H NMR spectra of compound **3d.**

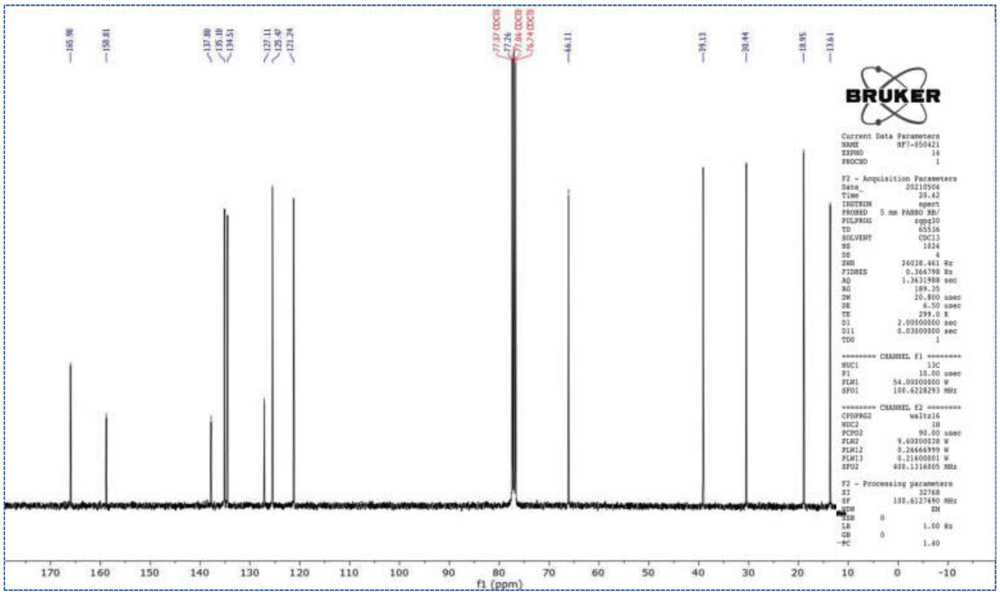

Figure S19. ^{13}C NMR spectra of compound **3d.**

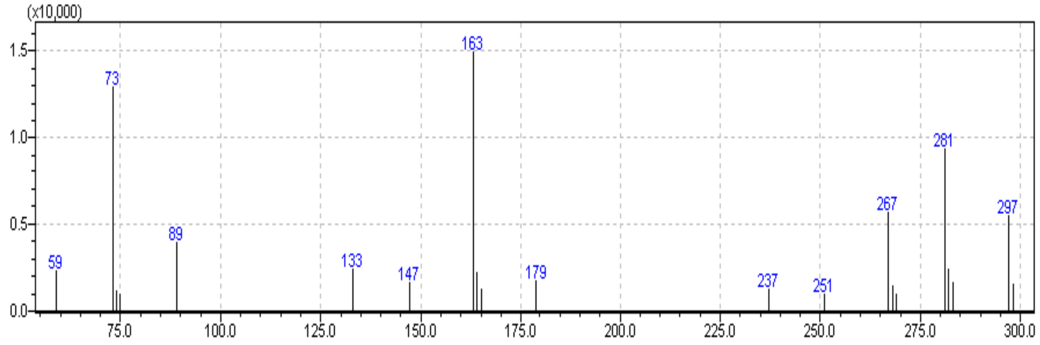

Figure S20. MS spectra of compound **3d.**

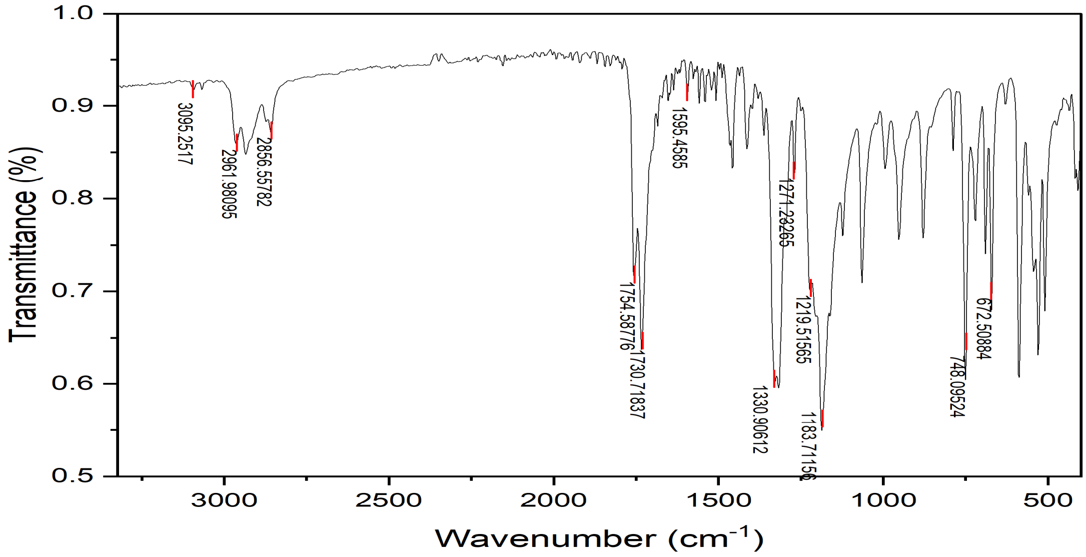

Figure S21. IR spectra of compound **3e.**

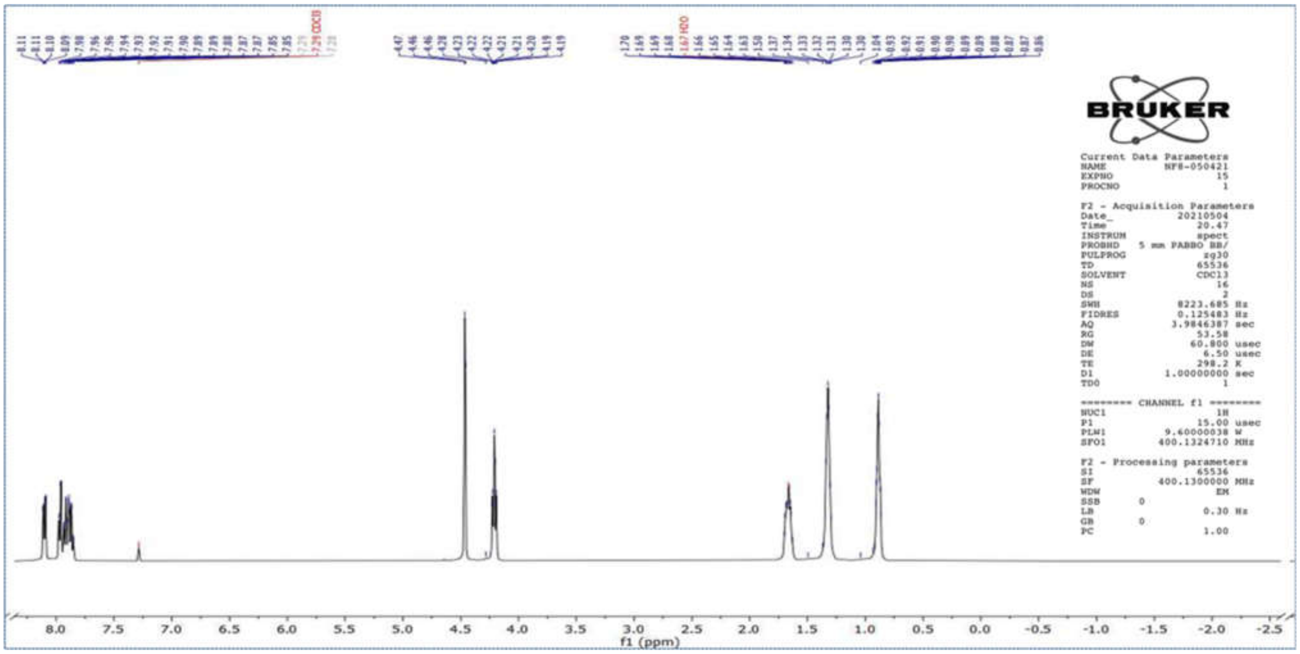

Figure S22. ^{1}H NMR spectra of compound **3e.**

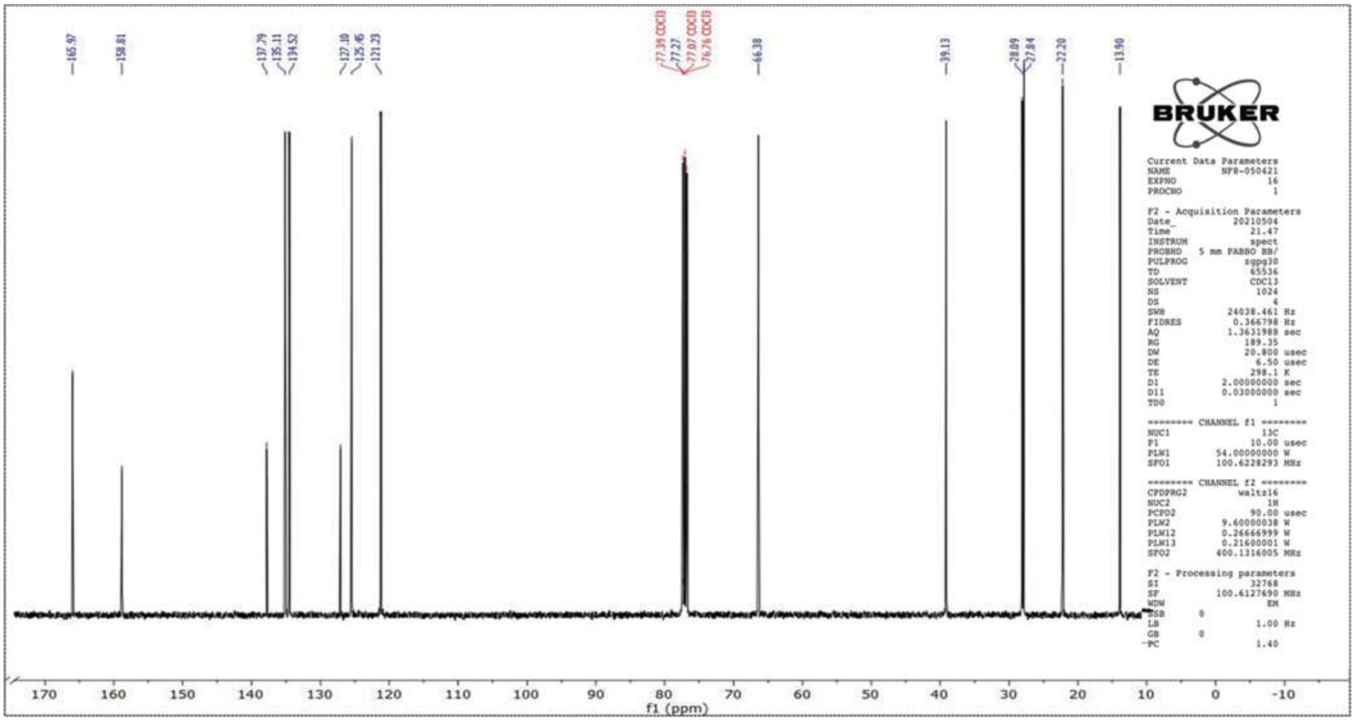

Figure S23. ^{13}C NMR spectra of compound **3e.**

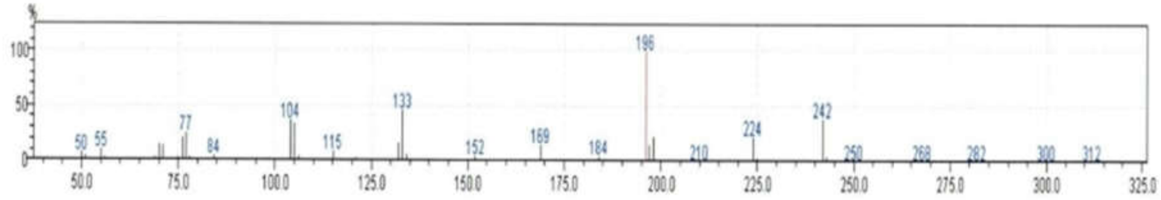

Figure S24. MS spectra of compound **3e.**

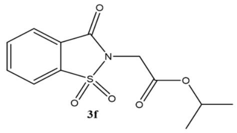

3f

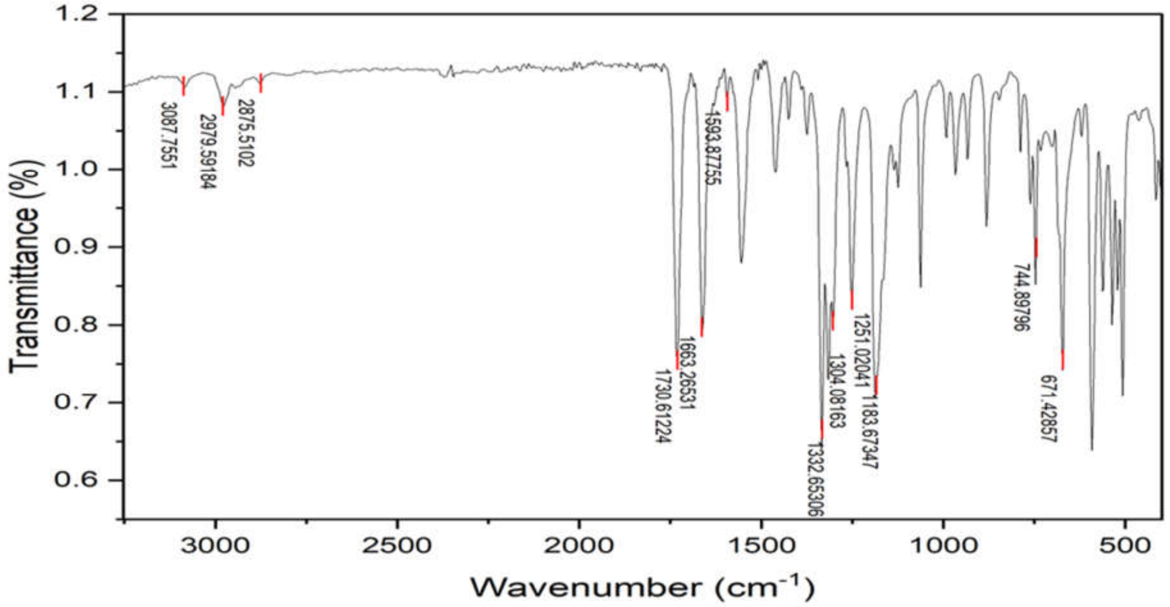

Figure S25. IR spectra of compound **3f**.

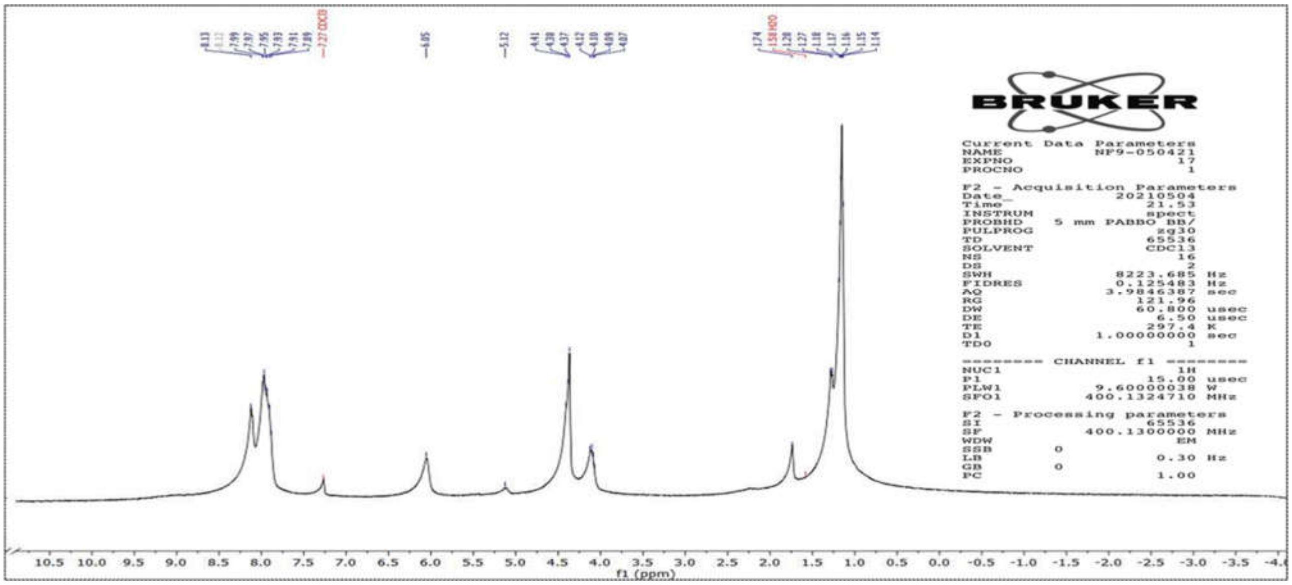

Figure S26. 1H NMR spectra of compound **3f**.

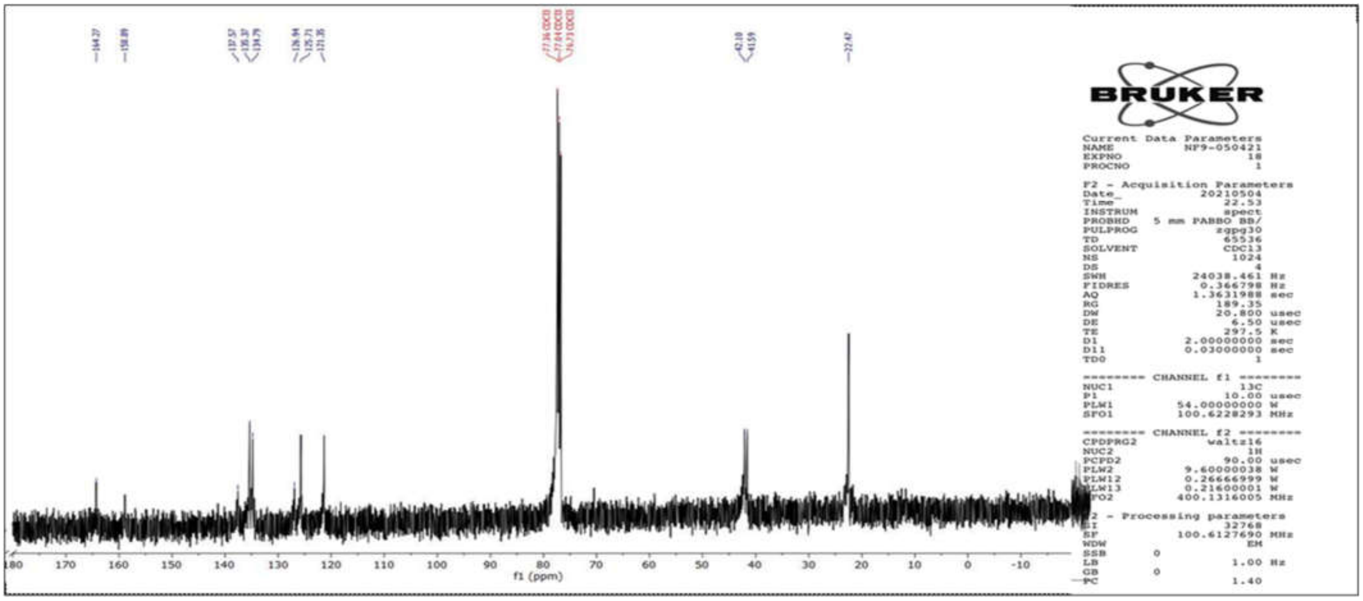

Figure S27. ^{13}C NMR spectra of compound **3f**.

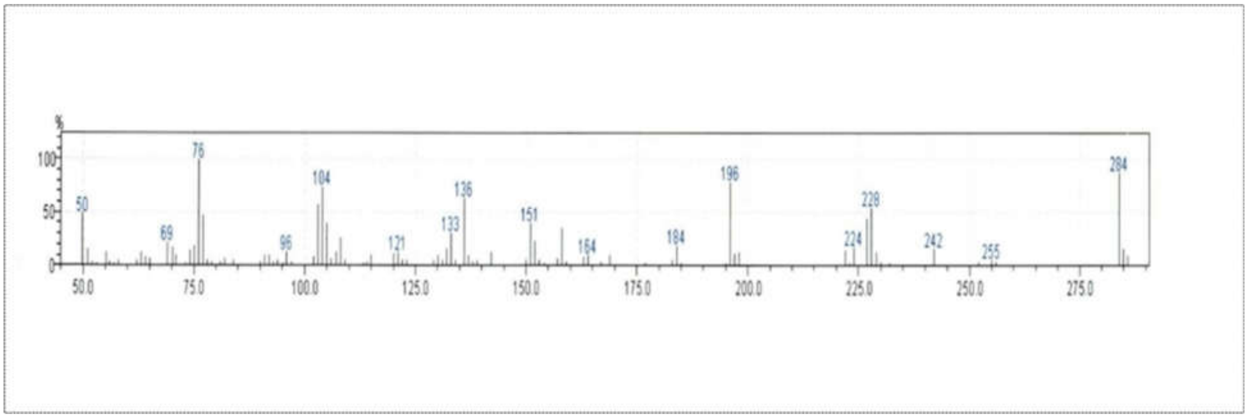

Figure S28. MS spectra of compound **3f**.

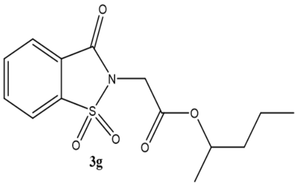

3g

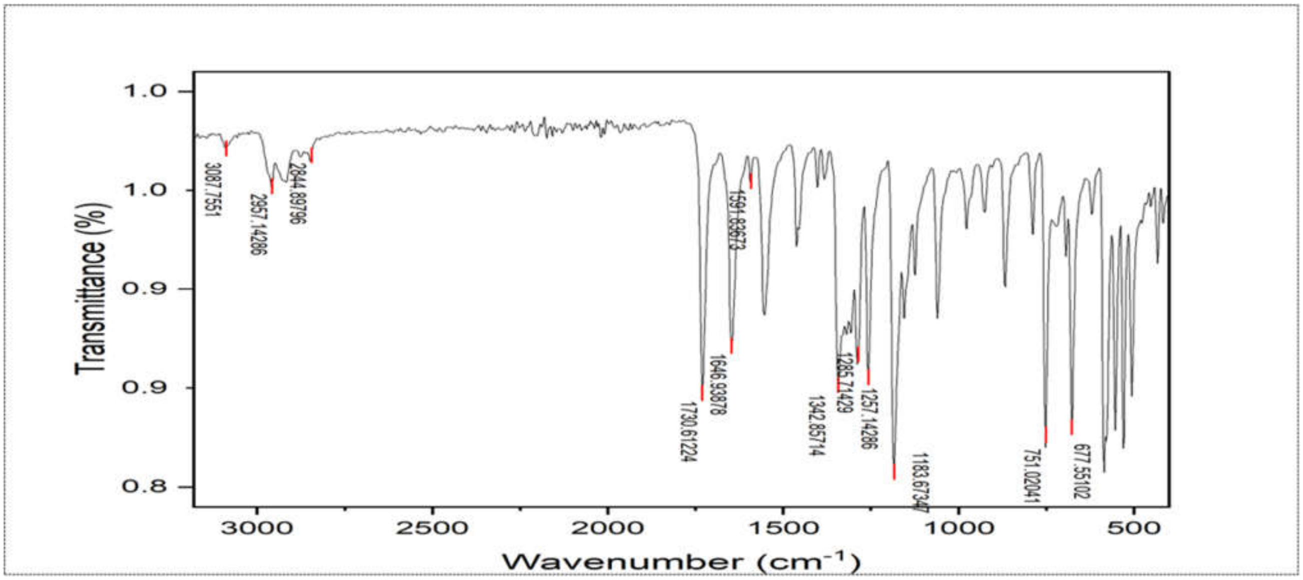

Figure S29: .IR spectra of compound **3g**.

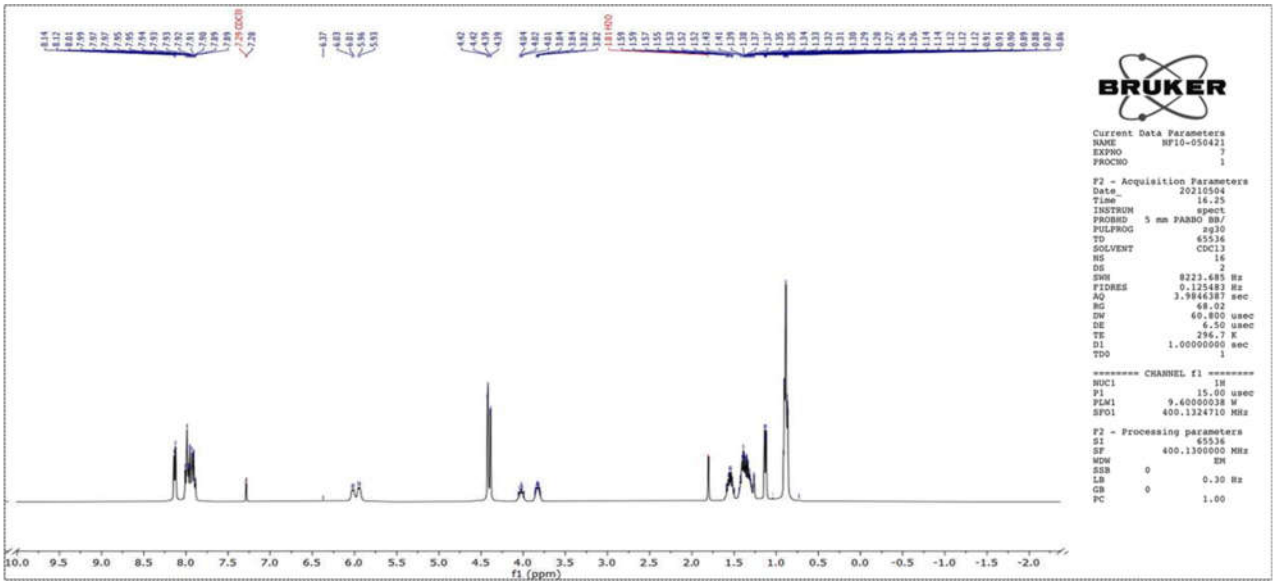

Figure S30. 1H NMR spectra of compound **3g**.

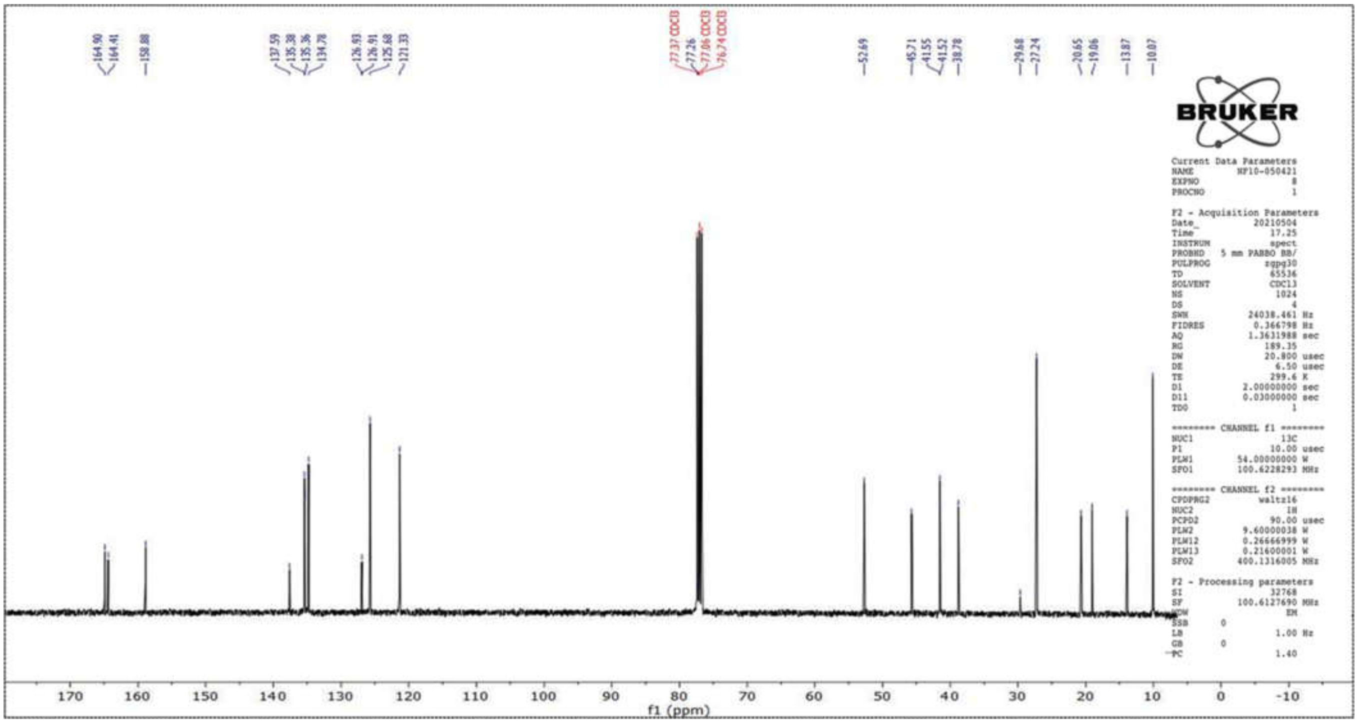

Figure S31. ^{13}C NMR spectra of compound **3g**.

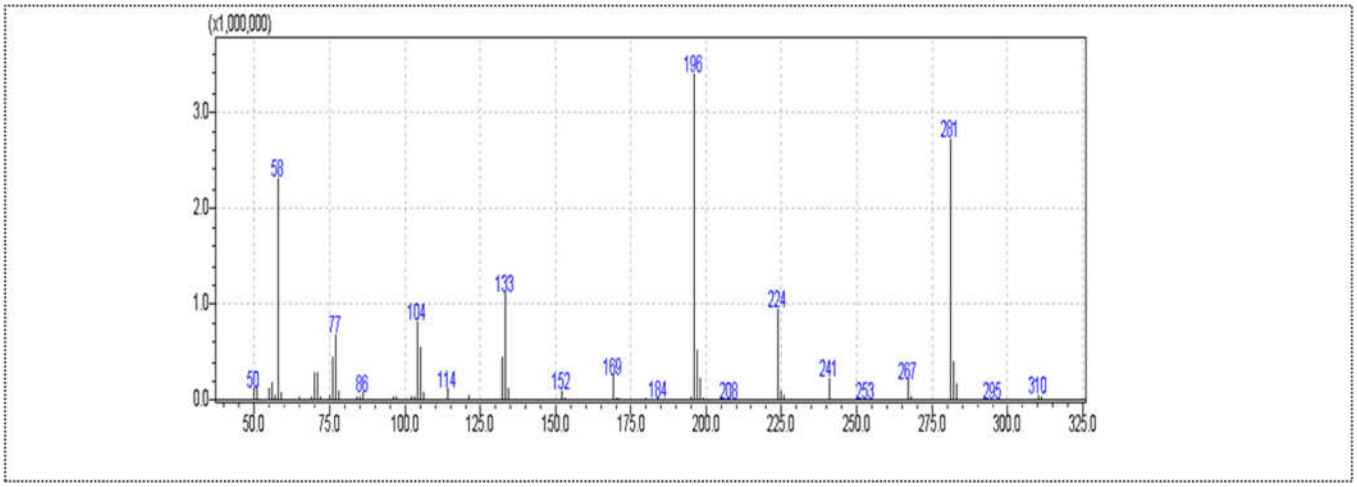

Figure S32. MS spectra of compound **3g.**

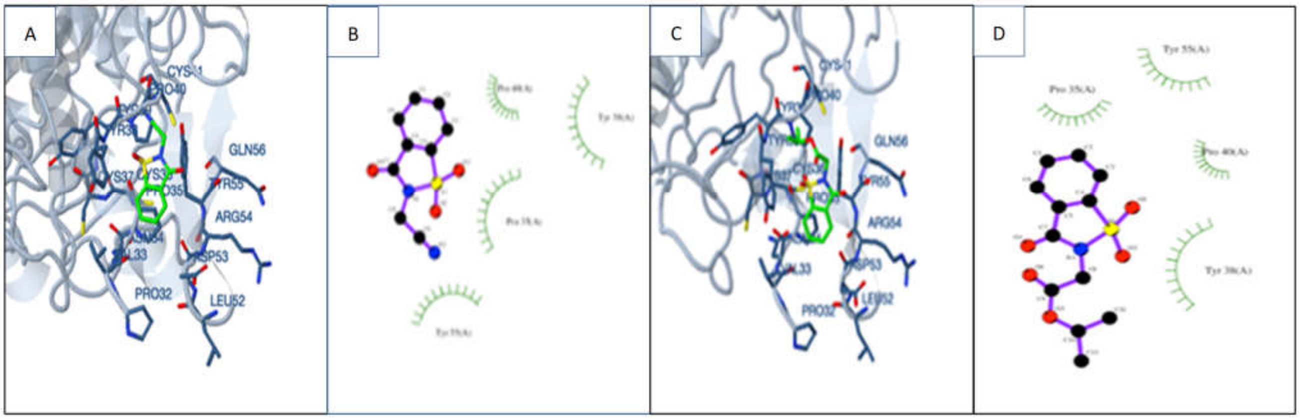

Figure S33. Molecular docking studies; A, B 3D, and 2D representations for compound **2**, respectively; C, D 3D, and 2D representations for compound **3f**, respectively; Hydrogen bonds highlighted in green with the respective distances between the atoms involved, whereas polar, hydrophobic, and other bonds are represented by an arc with spokes radiating towards the ligand atom.

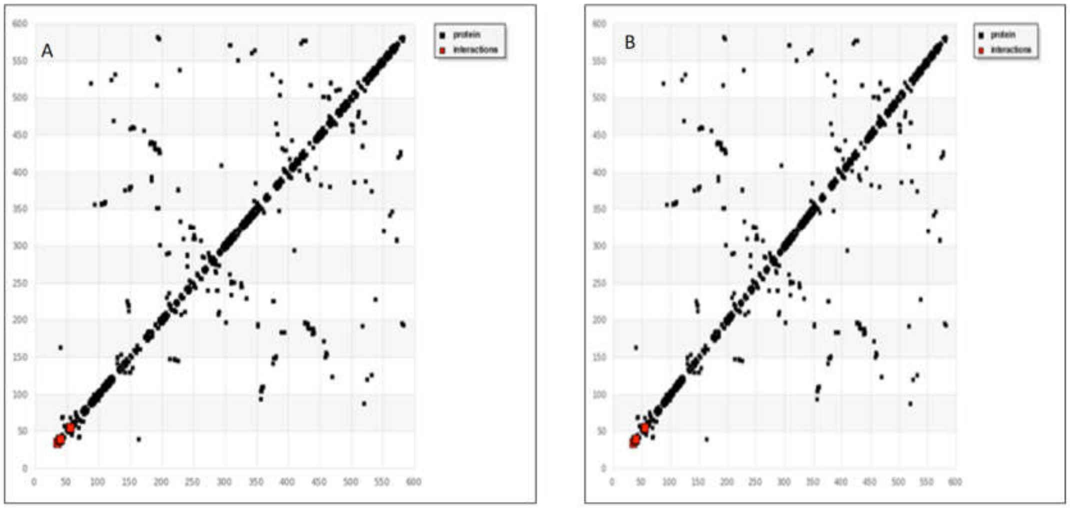

Figure S34. A, B, and C HB plot representations for compound **2**, and 3**f**, respectively docking against COX-1 enzyme.

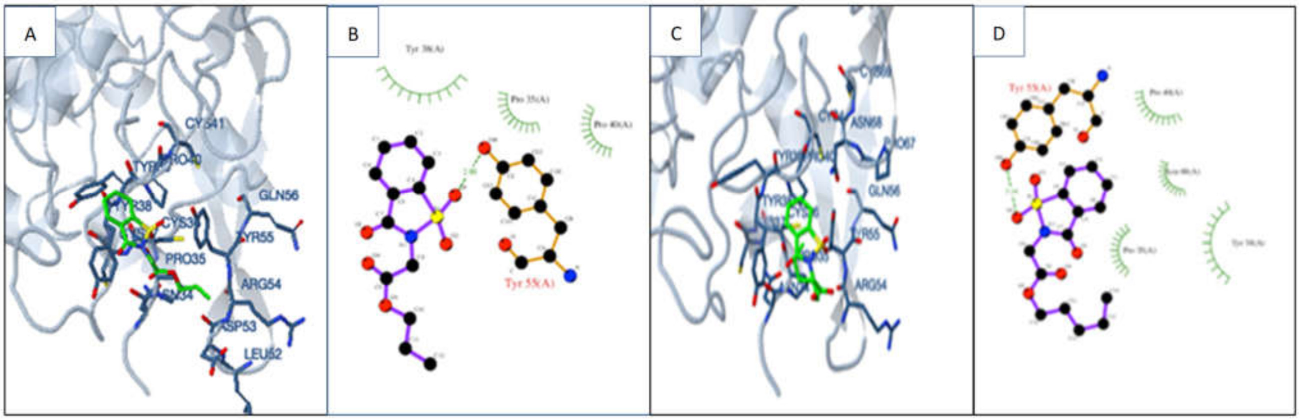

Figure S35. Molecular docking studies; A, B 3D, and 2D representations for compound **3C**, respectively; C, D 3D, and 2D representations for compound **3e**, respectively; Hydrogen bonds highlighted in green with the respective distances between the atoms involved, whereas polar, hydrophobic, and other bonds are represented by an arc with spokes radiating towards the ligand atom.

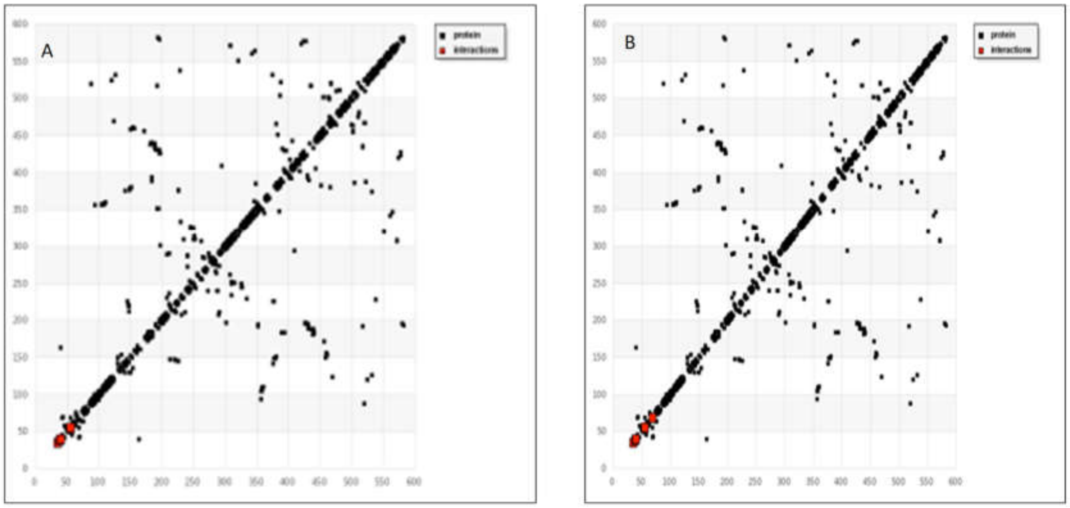

Figure S36. A, B, and C HB plot representations for compound **3c**, and 3e, respectively docking against COX-1 enzyme.

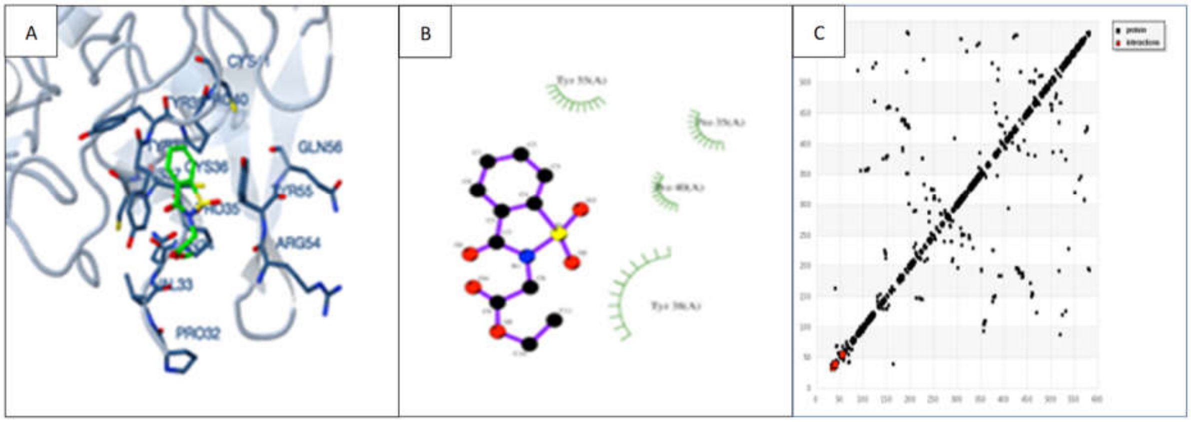

Figure S37. Molecular docking studies; A, B 3D, and 2D representations for compound **3b**, respectively; Hydrogen bonds highlighted in green with the respective distances between the atoms involved, whereas polar, hydrophobic, and other bonds are represented by an arc with spokes radiating towards the ligand atom. C HB plot representations for compound **3b** docking against COX-1 enzyme.

Table S1. MTT assay on normal cell lines (WI138).

Compounds (50µmol)	Number of dead cells(cells/ml)	Inhibition rate (%)
**2**	17	42.5
**3a**	4	10
**3b**	3	7.5
**3c**	11	27.5
**3d**	2	5
**3e**	15	37.5
**3f**	14	35
**3g**	16	40
